# Supplementary material for: Magnetostriction-polarization coupling in multiferroic Mn2MnWO6
Source: Nat Commun. 2017 Dec 11;8:2037. doi: 10.1038/s41467-017-02003-3 (PMC5725588; doi:10.1038/s41467-017-02003-3)
Supplement: Supplementary file 1 — Supplementary Information [file 41467_2017_2003_MOESM1_ESM.pdf]

Supplementary Figures

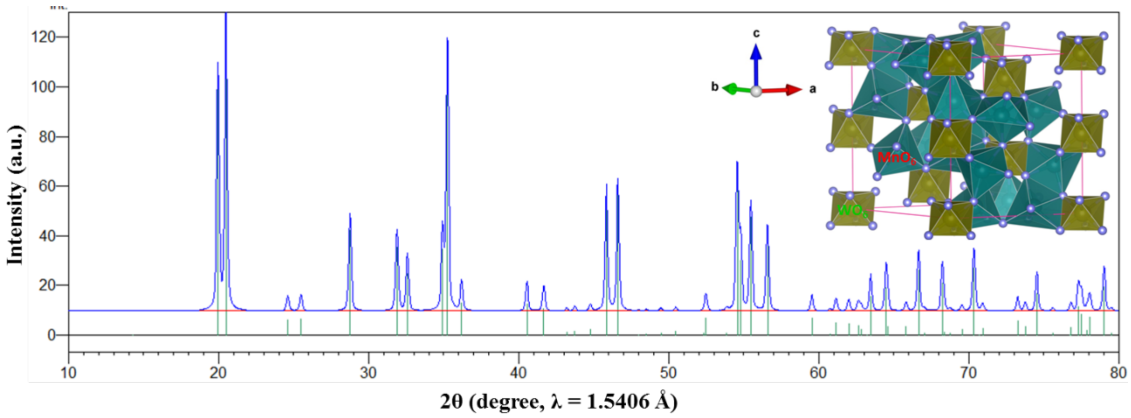

Supplementary Figure 1 | Simulated lab XRD patterns and crystal structure of Mg_3TeO_6-type Mn_2MnWO_6. Crystallographic data: *R*-3, *a* = 8.8931(18) Å, *c* = 10.4782(19) Å, *V* = 717.7(1) $Å^3$. Inset shows the polyhedral crystal structure with octahedral coordination of Mn and W.

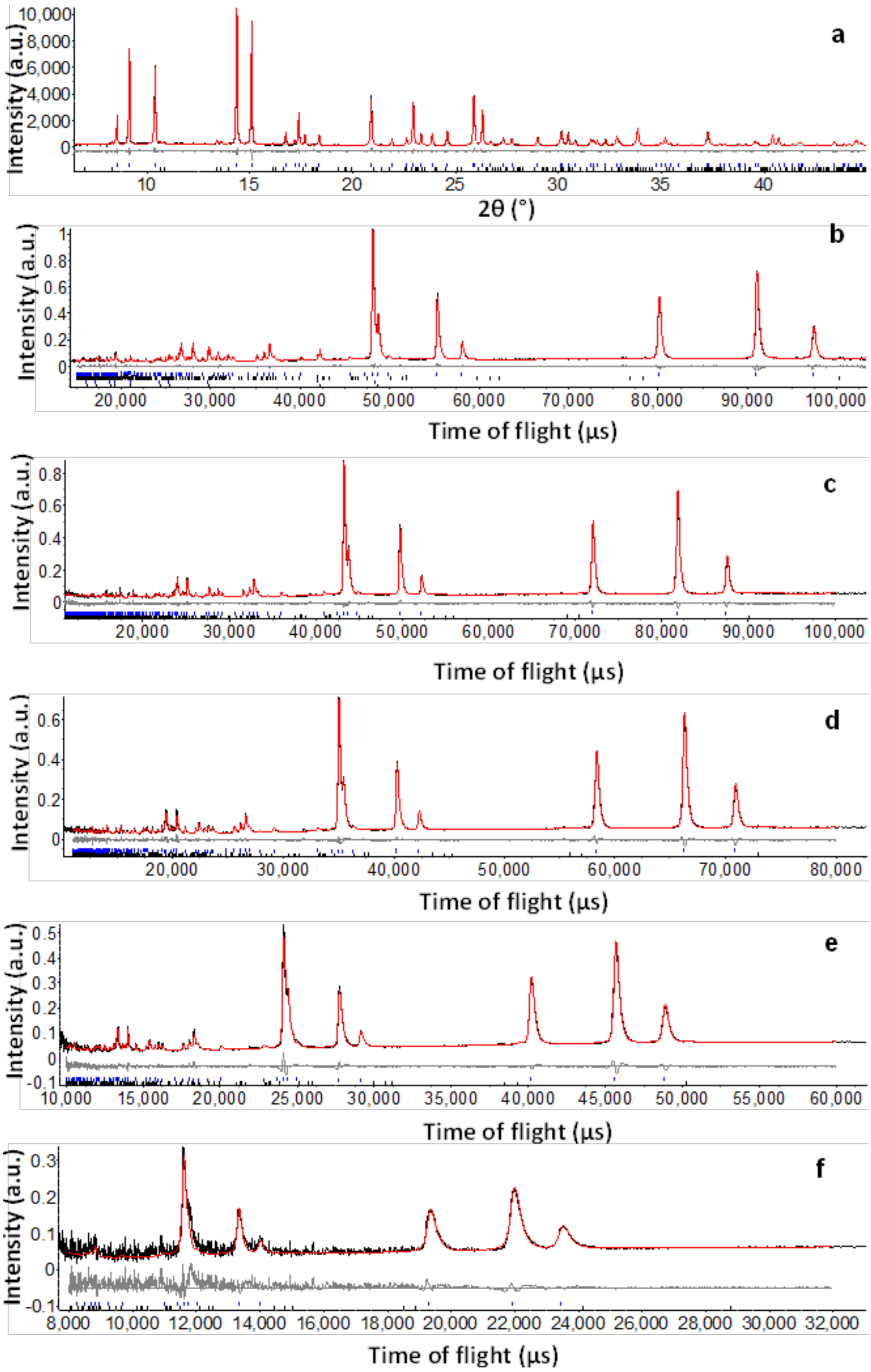

Supplementary Figure 2 | Rietveld refinement profiles for Mn_2MnWO_6 at 290 K. a SPXD data **b** high resolution bank 5 (153°, ~0.7 – 4.8 Å) NPD data, **c** bank 4 (122°, ~ 0.7 – 5.4 Å) NPD data, **d** bank 3 (90°, ~ 0.7 – 6.6 Å) NPD data, **e** bank 2 (58°, ~ 1 – 9.6 Å) NPD data and **f** bank 1 (27°, ~ 1.6 – 17 Å) NPD data. Observed, calculated and difference profiles are shown by black points and with red and gray lines, respectively. Tick marks show the peak positions for the main phase (top) and impurity phase $MnWO_4$ (3.3(1)% by weight). For bank 5 data two Pawley phases were also included in the refinement to fit peaks from Al (~42000 μs, ~30000 μs, ~25000 μs) due to scattering from the cryostat.

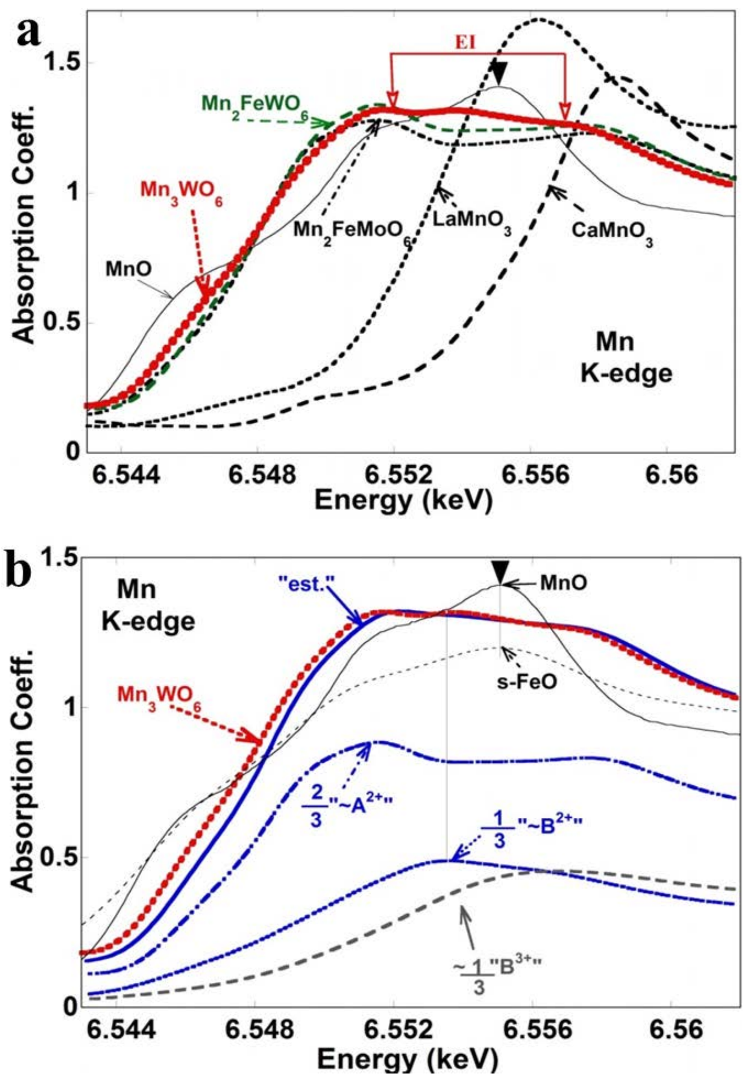

Supplementary Figure 3 | The Mn-K edge XANES of Mn_2MnWO_6. **a** Spectra for Mn_2MnWO_6, the isostructural $Mn_2Fe\mathit{T}O_6$ (T = W and Mo) compounds, along with those of a series of standard compound spectra: $Mn^{2+}O$, $LaMn^{3+}O_3$, and $CaMn^{4+}O_3$. The energy range, labeled EI, where the Mn_2MnWO_6 exhibits extra intensity is relative to the T = W and Mo compounds. Note the large arrow indicating the calibration energy at the peak of the MnO spectrum. **b** The Mn-K edge spectrum for Mn_2MnWO_6. The 2/3 $\sim A^{2+}$ component was estimated from the 2/3 weighting of the isostructural (and iso-valent) Mn_2FeWO_6 Mn-K edge. Note the s-FeO spectrum is the FeO, Fe-K edge shifted down in energy so that its peak coincides with the MnO, Mn-K edge peak. The 1/3 $\sim B^{2+}$ and 1/3 $\sim B^{3+}$ spectral component estimates were obtained by applying this same energy shift to the Fe-K edges of $Mn_2Fe^{\sim 2+}WO_6$ and $Mn_2Fe^{\sim 3+}MoO_6$ respectively (with the 1/3 weighting factor). The $\sim B^{3+}$ has its spectral intensity lying at too high energy to consider it as a contributing component to the Mn_2MnWO_6 spectrum. The estimated spectrum (labeled est. in the figure) is a superposition of the 2/3 $\sim A^{2+}$ and 1/3 $\sim B^{2+}$ components.

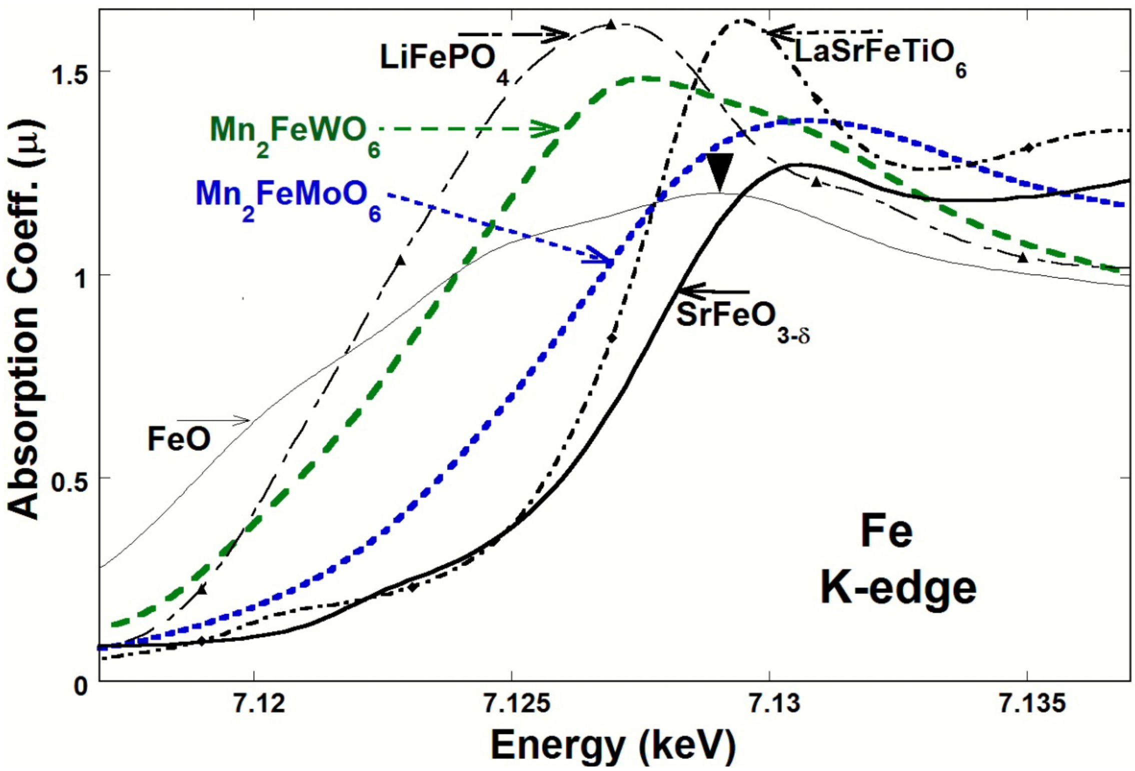

Supplementary Figure 4 | The Fe-K edge XANES of $Mn_2Fe^{2+}WO_6$ and $Mn_2Fe^{3+}MoO_6$ compounds. Along with those of a series of standard compound spectra are also shown: $Fe^{2+}O$, $LiFe^{2+}PO_4$, $LaSrFe^{3+}TiO_6$, and $SrFe^{\sim 4+}O_{3-\delta}$. It is important to note the very substantial broadening of the Fe-K edge spectra at the B site of these $A_2BB'O_6$ materials with the Ni_3TeO_6 (NTO, *R*3) structure. Despite this broadening the chemical shift between the $Fe^{2+}$and Fe^{3+} B-site spectra is very clear. The Fe-K $Mn_2Fe^{2+}WO_6$ spectrum, relative to the FeO peak energy, is used to estimate the B^{2+}-site contribution for Mn_3WO_6 in the main portion of this work. Note the large arrow indicating the calibration energy at the peak of the FeO spectrum.

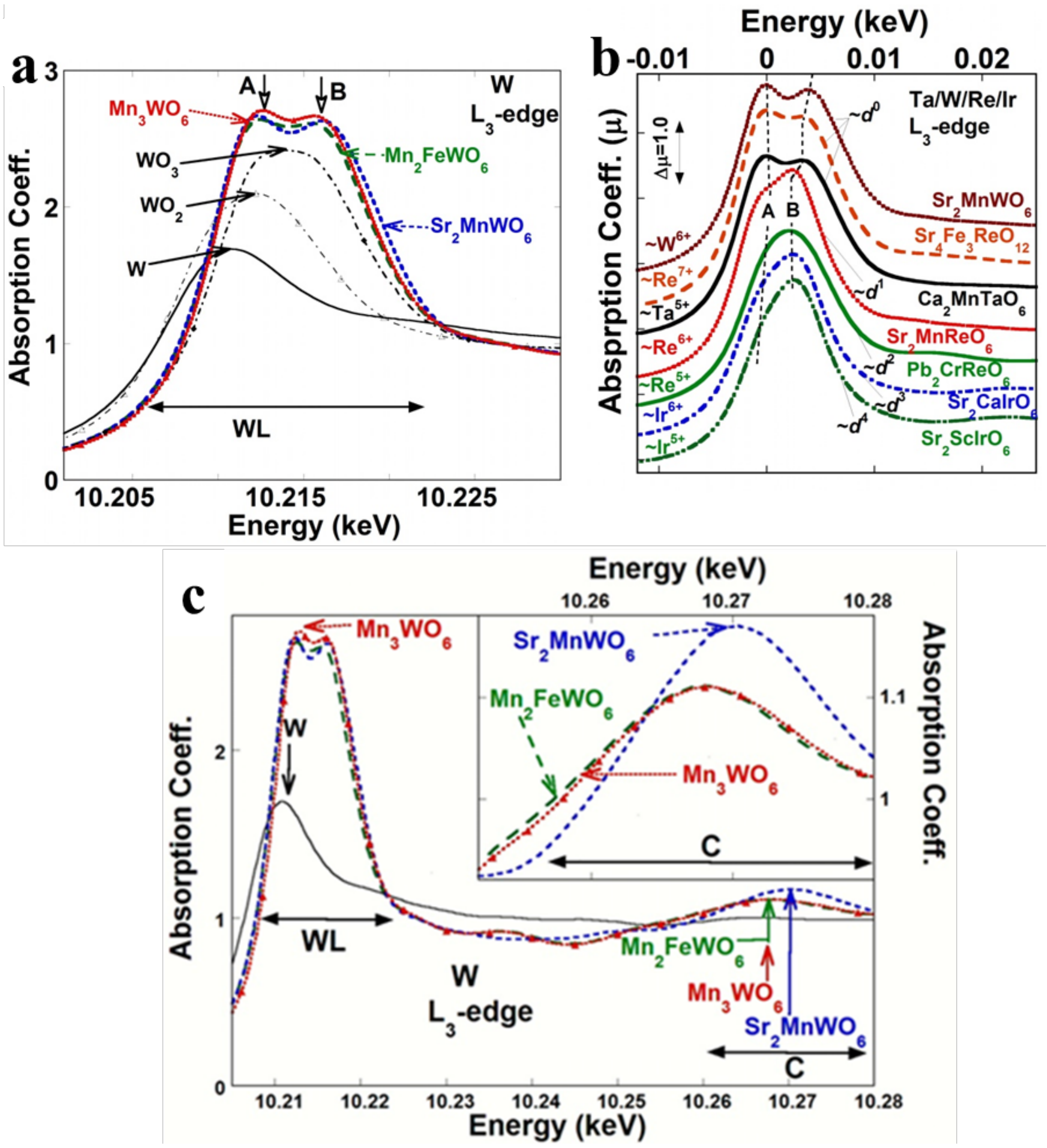

Supplementary Figure 5 | The W-L_3 edge XANES of Mn_2MnWO_3. a Comparison of the series of W standard compounds in various d-configurations/valence states: the ~ d^0-W^{6+} compound Sr_2MnWO_6, Mn_2FeWO_6 and WO_3; the ~ d^2-W^{4+} compound WO_2; and elemental W. Note the systematic white line WL chemical shift to higher energy with increasing nominal W valence. Note also the bimodal A-B structure of WL-5*d* features in the W^{6+} compounds strong octahedral ligand field splittings. **b** The T-L_3 edges for a series of T(5*d*) compounds in various *d*-configurations/valence states from ~ d^0 ($t_{2g}{}^0$) to ~ d^4 ($t_{2g}{}^4$). **c** The W-L_3 edge XANES spectra for Mn_2MnWO_6, Mn_2FeWO_6, and Sr_2MnWO_6 shown on an energy range encompassing both the WL and the prominent fine structure C-feature regions. Inset shows the C-feature region on an expanded scale.

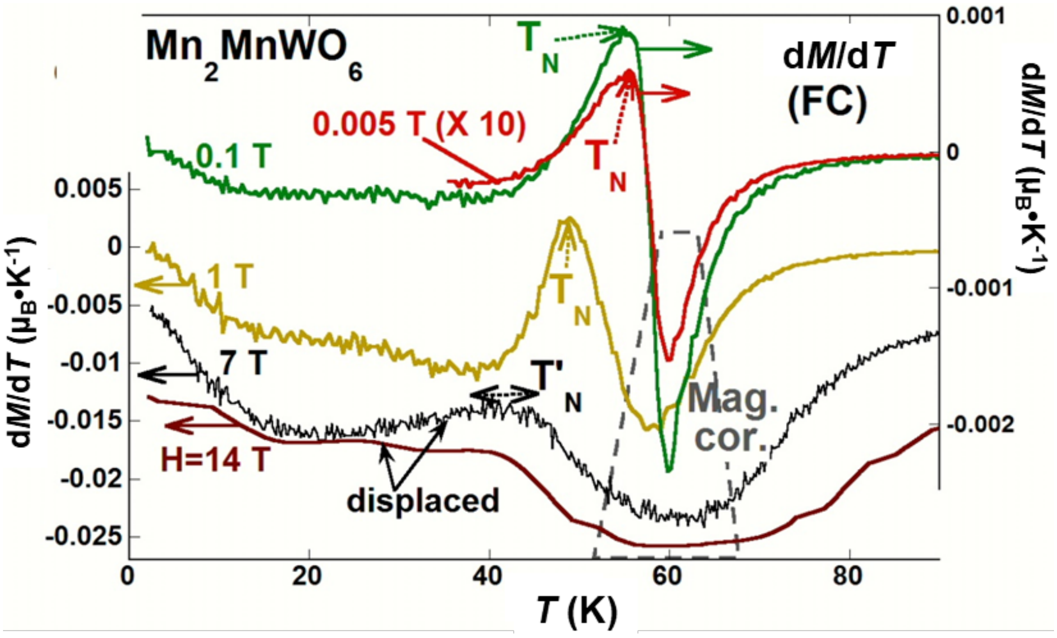

Supplementary Figure 6 | The dM/dT derivatives for the FC and M(T) curves in Fig. 4b at a series of magnetic fields. For this comparison, the high field and low field plots are displayed on different vertical scales, and the H = 0.005 T curve is multiplied by a factor 10. Note the sharp positive peaks indicating the magnetic ordering temperatures T_N, for $H \leq 1$ T. The T_N value at 0.005 T is 55.4 K in excellent agreement with the neutron diffraction results (see **Supplementary Figure 11** caption). The similar broadened structure, labelled T_N' for the H = 7 T curve, is interpreted here as tentative evidence for the presence of AFM order/correlations. Note the negative dM/dT peaks in the ~ 60 K range at all fields, are associate magnetic correlations above the ordering temperature. For $H \leq 1$ T these negative peaks are quite sharp, however at higher fields they are strongly broadened. The negative dM/dT peaks and their widths (from **Supplementary Figure 6**) define a dash line boxed range where these field-coupled magnetic correlations (Mag. cor. in figure) appear operative.

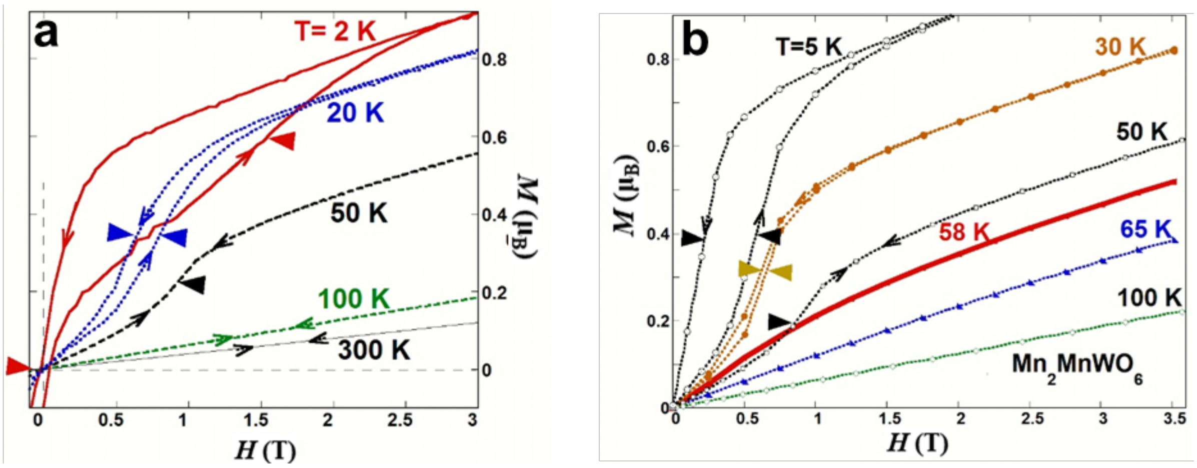

Supplementary Figure 7 | Expanded views of the positive field portion of the *M*(*H*) loops. It is for this system made in different laboratories and with differing maximum field in the loop. **a** An expanded view of the data shown in **Fig. 2c** of the main text. **b** An expanded view of magnetization loop data collected between ± 3.7 T, and except for the 100 and 50 K loops which were extended to ± 7 T. The solid horizontal arrowheads indicate the inflection point associated with the field induced transitions. Noting that the detailed choice of temperature/field history will lead to differing phase admixtures and metastability limits in field induced transitions, the two sets of data are in admirable agreement. The presence of 1st-order/hysteretic field induced transitions for $T \leq 30$ K, along with an apparently continuous (2nd-order) at $T = 50$ K, is supported by the data. We note the slight offset in *H* at the start of the 2 K field sweep measurement presumably caused by some remnant field at the start of this measurement, rather than starting in a true zero field state.

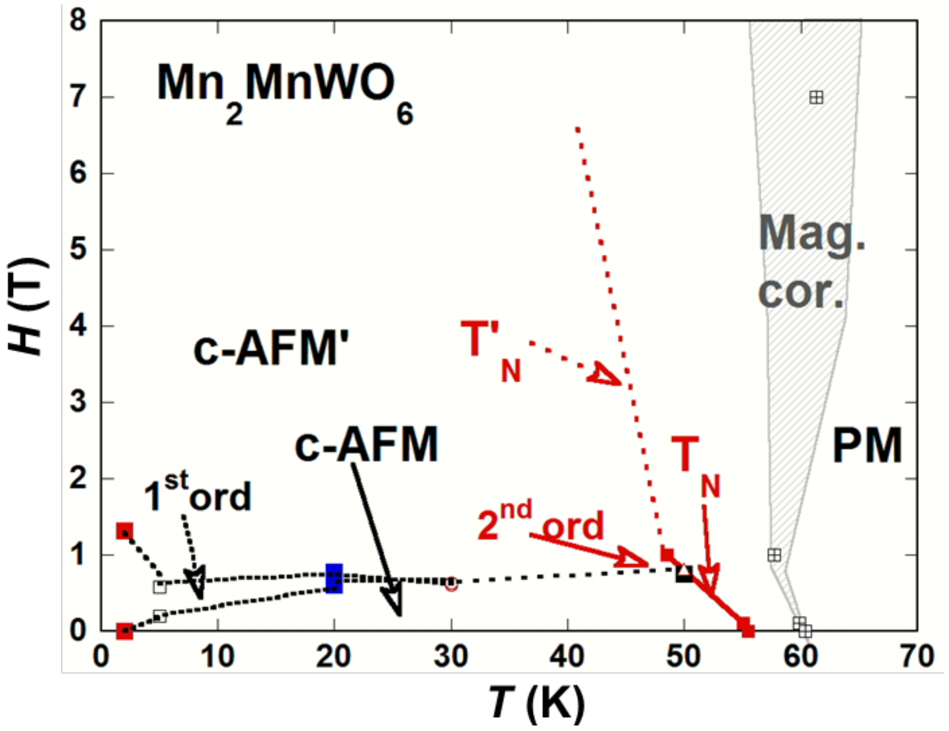

Supplementary Figure 8 | A schematic low temperature *H*-*T* phase diagram for Mn_2MnWO_6 based upon the magnetic property measurements. The solid red line labelled T_N is the onset of a c-AFM ordered state as indicated by the inflection point below the peak in the *M*(*T*) data in **Fig. 2**b and the corresponding sharp positive d*M*/d*T* peaks in **Supplementary Figure 6**. Here the c- prefix to conical-AFM conveys the conical and complex nature of the ordering found from the neutron scattering. The higher field dotted line (labeled T_N') indicates the possible onset of a more different c-AFM' state with a larger magnetic field response. In view of crystalline magnetic anisotropies, and the polycrystalline nature of the samples, any detailed comments on this phase would be speculative without finite -field neutron diffraction results. Also, the region labelled Mag. cor. indicates region where presumably local magnetic correlations, coupled to the magnetic field appear operative. The long range character in this region remains paramagnetic (PM).

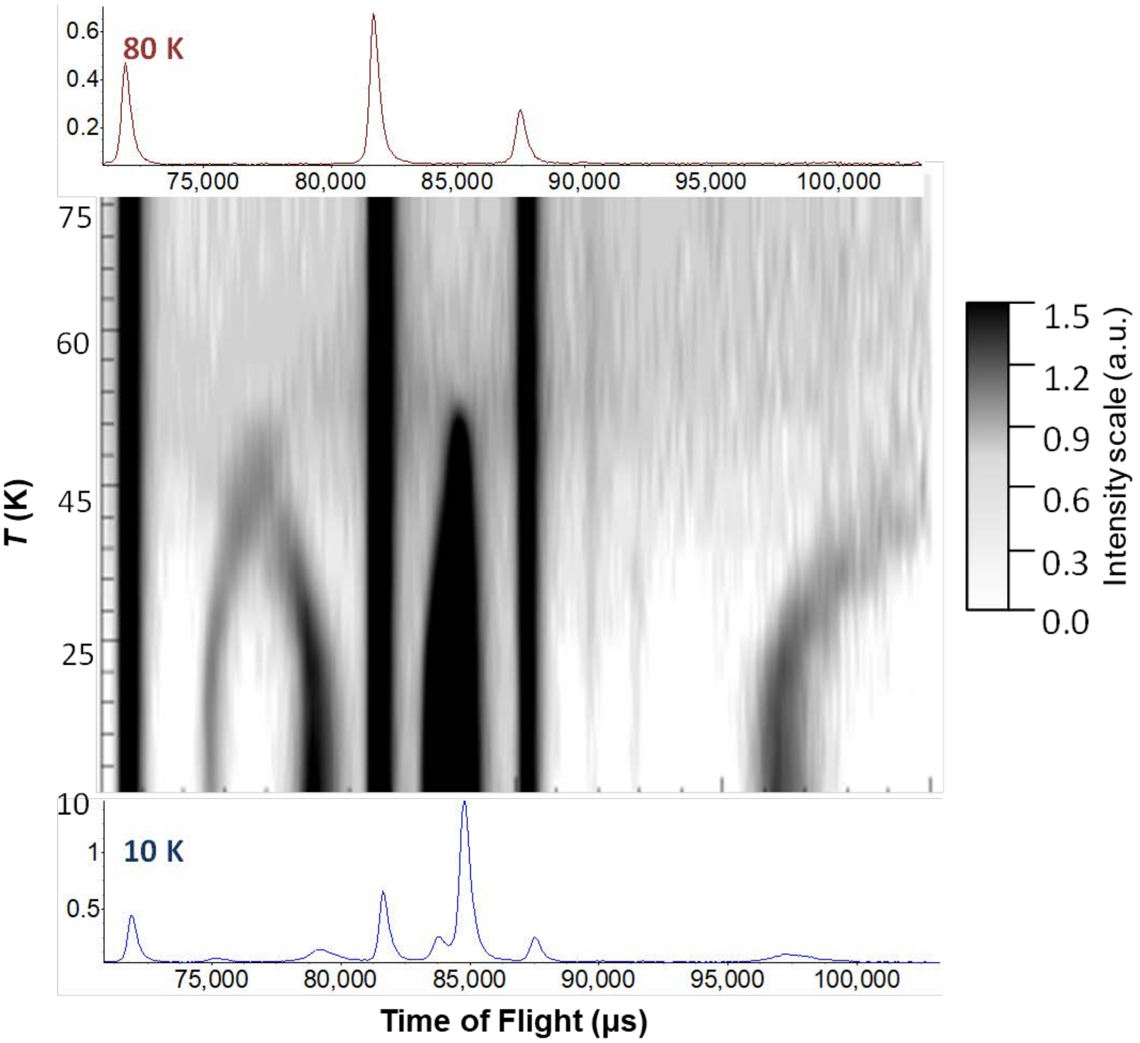

Supplementary Figure 9 | Film plot showing high d spacing region of bank 4 NPD data. They show intensity of commensurate (~ 85000 μs, ~4.6 Å) and incommensurate (~75000 μs (4.0 Å), ~79000 μs (4.2 Å) and ~97000 μs (5.2 Å)) magnetic reflections.

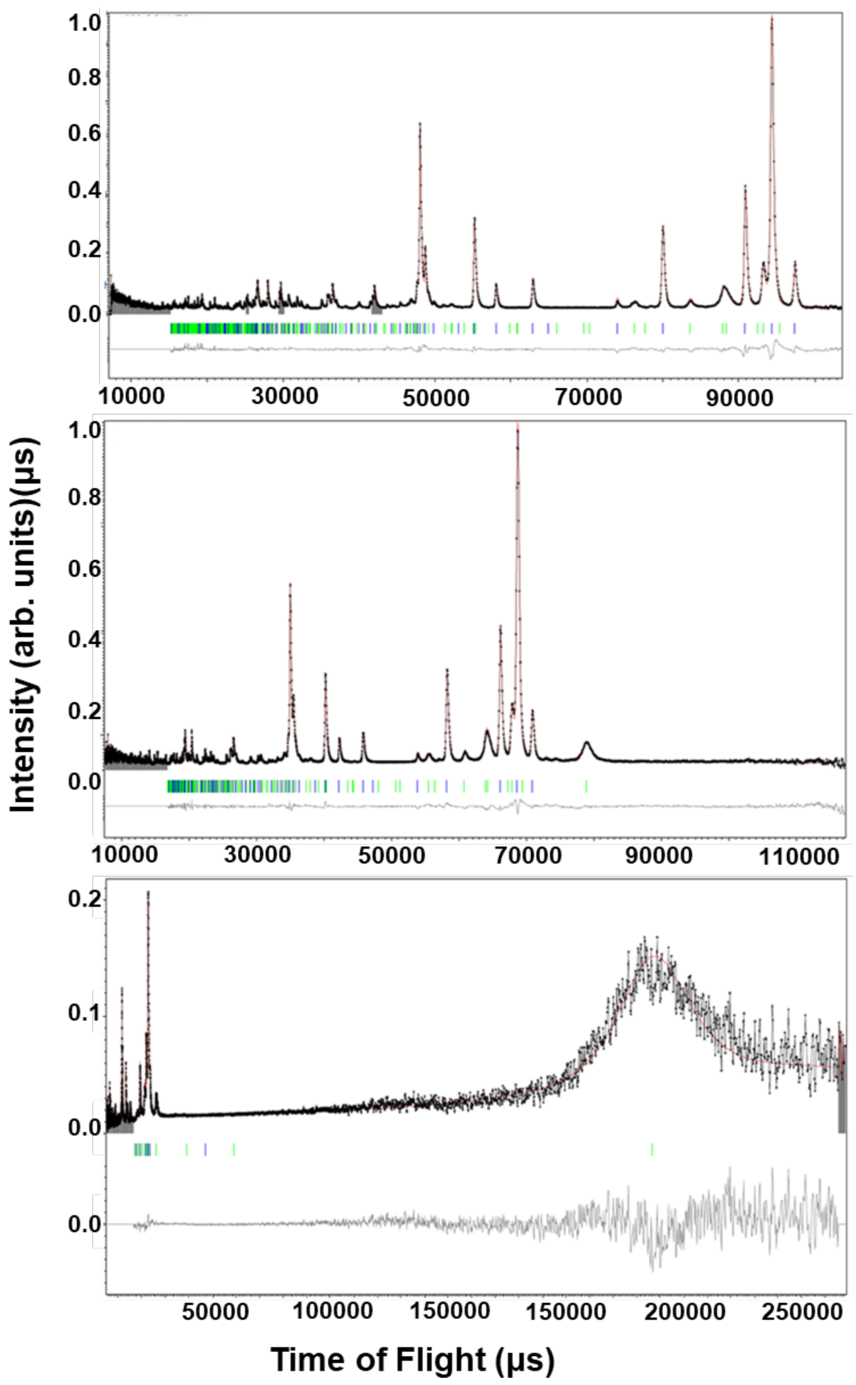

Supplementary Figure 10 | Rietveld refinement profiles for 5 K NPD data for Mn_2MnWO_6. **a** High resolution bank 5 (153°, ~0.7 – 4.8 Å) data; **b** bank 3 (90°, ~0.7 – 6.6 Å) data and **c** double-frame bank 1 (27°, ~1.7 – 60 Å) data showing magnetic reflection at ~47 Å (~235000 μs). Observed, calculated and difference profiles are shown by black points and with red and grey lines, respectively. Blue and green tick marks show Mn_2MnWO_6 commensurate and satellite peak positions, respectively. Excluded regions are shaded in grey, including peaks due to Al (~ 2000, ~ 30000, ~ 25000 μs) due to scattering from the cryostat in bank 5.

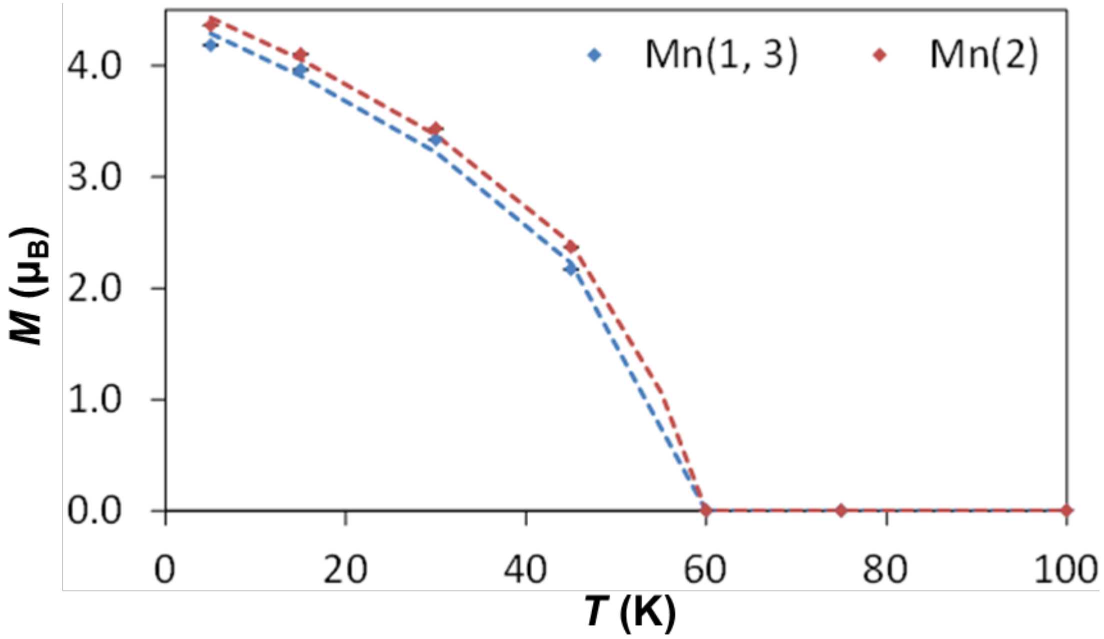

Supplementary Figure 11 | Magnetic moments for Mn_2MnWO_6 as a function of temperature from Rietveld refinements (refinements using Jana2006 with bank 5 (153°), bank 3 (90°) and bank 1 (27°) data). The Mn_2MnWO_6 structure was described by a model of $R_I3(00g)t$ (hexagonal setting) symmetry with commensurate nuclear structure and incommensurate magnetic structure with moments on all Mn sites described by both mT1 and mΛ2LE2 irreps. Data points for Mn(1)/(3) sites are shown in blue and red (with esds approximately the same size as the points) and dotted lines are a guide to the eye showing fit to function $M_T = M_0\left[1-\left(\frac{T}{T_N}\right)\right]^{\beta}$ where M_T is the sum of intensities at temperature T; for Mn(1)/(3): M_0 = 4.46(7), T_N = 55.7(1) K and β = 0.42(2); for Mn(2): M_0 = 4.60(4), T_N = 56.6(2) K and β = 0.41(1).

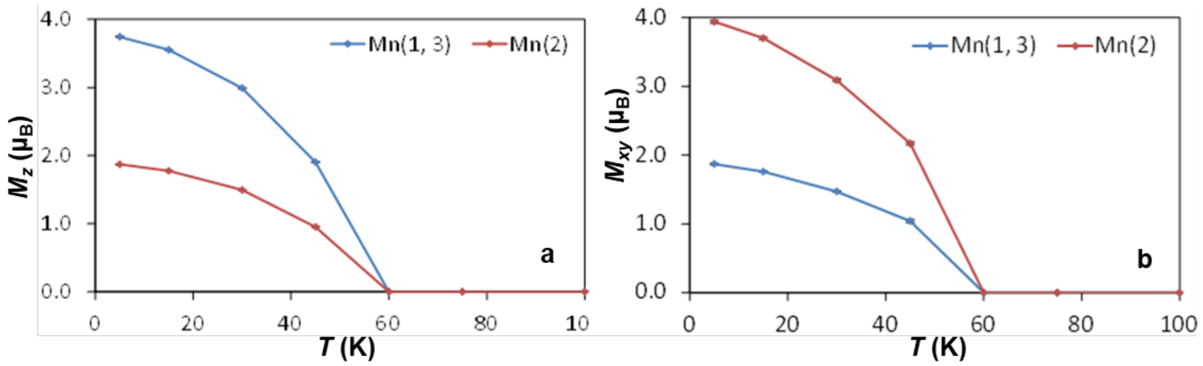

Supplementary Figure 12 | Components of magnetic moments for Mn_2MnWO_6 as a function of temperature from Rietveld refinements using NPD data. Data points for M_z(μ$_B$) for Mn1/3 and Mn2 (Left) and M_{xy}(μ$_B$) for Mn1/3 and Mn2 (Right) sites are shown in blue and red, respectively (with esds approximately the same size as the points).

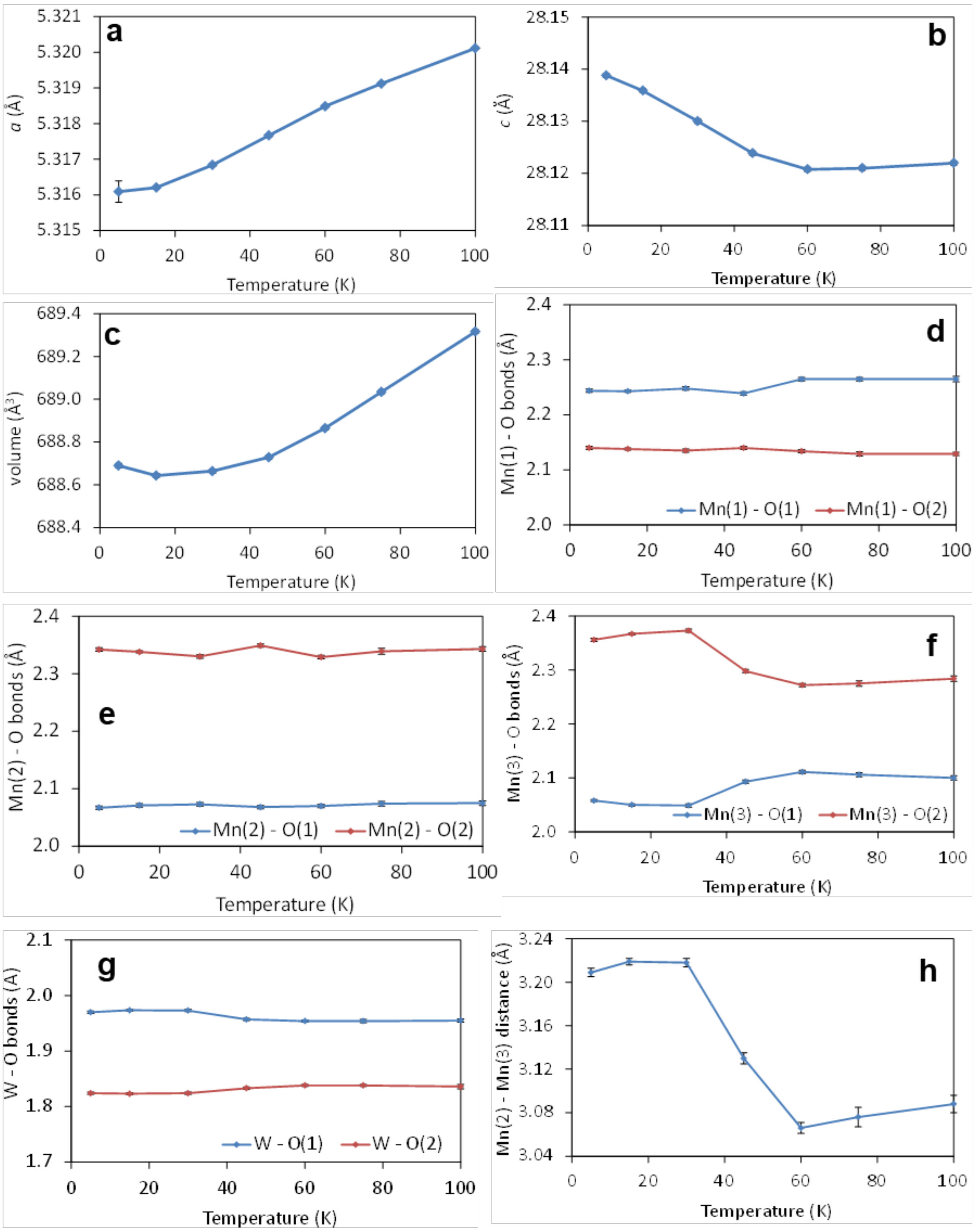

Supplementary Figure 13 | Unit cell parameters and selected bond lengths and distances from refinements using short NPD scans (refinements using Jana2006 with bank 5 (153°), bank 3 (90°) and bank 1 (27°) data). **a** *a*-*T*; **b** *c*-*T*; **c** *V*-*T*; **d** (Mn1-O)-*T*; **e** (Mn2-O) -*T*; **f** (Mn3-O)-*T*; **g** (W-O)-*T*; **h** (Mn2-Mn3)-*T*.The Mn_2MnWO_6 structure was described by a model of $R_I3(00g)t$ (hexagonal setting) symmetry with commensurate nuclear structure and incommensurate magnetic structure with moments on all Mn sites described by both mT1 and mΛ2LE2 irreps.

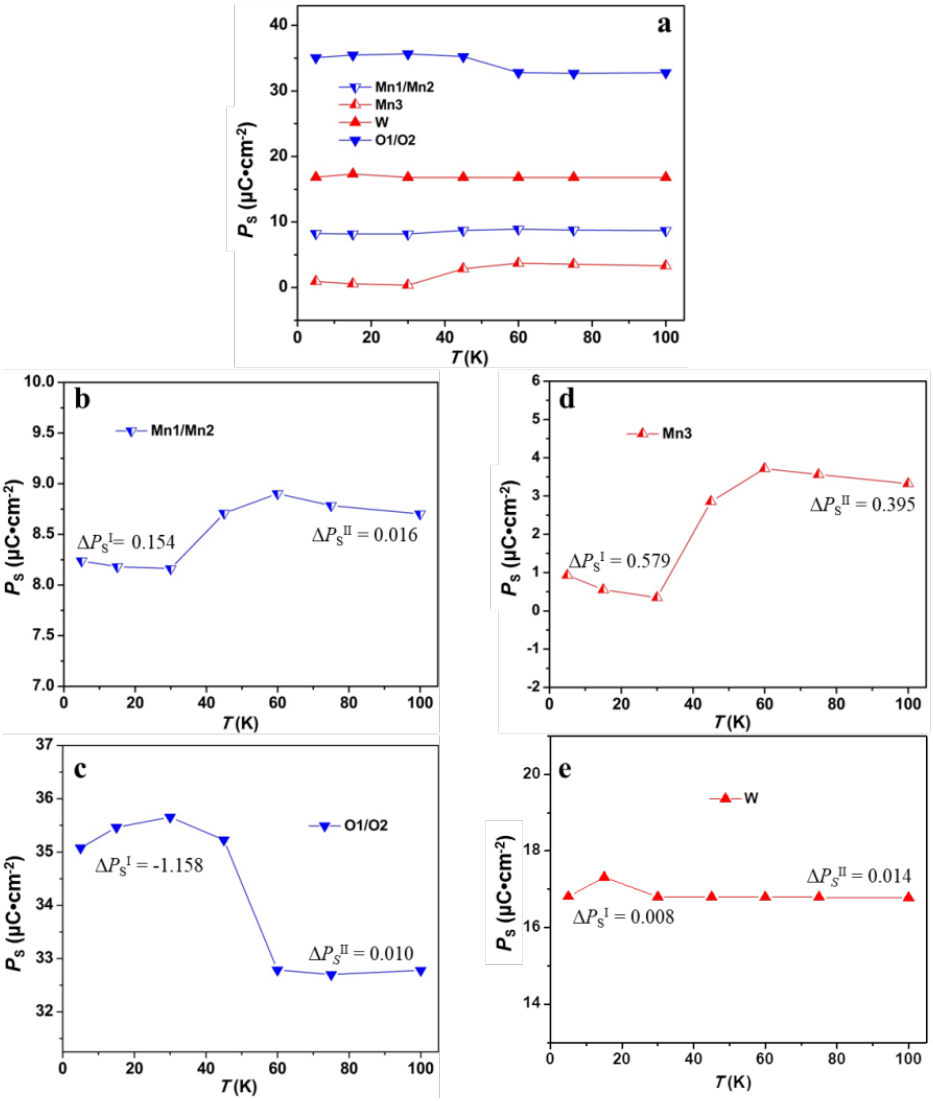

Supplementary Figure 14 | Temperature dependent individual atomic P_S contribution in Mn_2MnWO_6 between 5 and 100 K as calculated from point-charge-model method. The total net $P_S = P_S^+(\text{Mn1 + Mn2 + O1 + O2}) - P_S^-(\text{Mn3 +W})$ regarding the formal oxidation state and atomic displacement direction along the *c*-axis in **Fig. 1** (P_S^+(Mn1) = P_S^+(Mn2), P_S^+(O1) = P_S^+(O2) from point-charge-model calculations). The '+' and '-' represent the polarization directions denoted as blue (+) and red (-) symbols, respectively. ΔP_S^I and ΔP_S^{II} are for the P_S evolution between 5 and 30 K and 60 and 100 K upon cooling, respectively. P_S evolution of **a** Mn1/Mn2, O1/O2, Mn3, and W, and **b** Mn1/Mn2, (c) O1/O2, **d** Mn3, and **e** W. One should note that the polarization directions of (Mn1, Mn2, O1, O2) and (Mn3, W) are opposite. Apparently, the deceasing of ΔP_S^I(O1/O2) (1.158μC•cm^{-2}) and increasing of ΔP_S^I(Mn3) (0.579 μC•cm^{-2}) in opposite direction are mainly responsible for the total P_S drop of 1.59 μC•cm^{-2} between 5 and 300 K. The P_S values are almost identical between 60 and 100 K (0.01 μC•cm^{-2} difference).

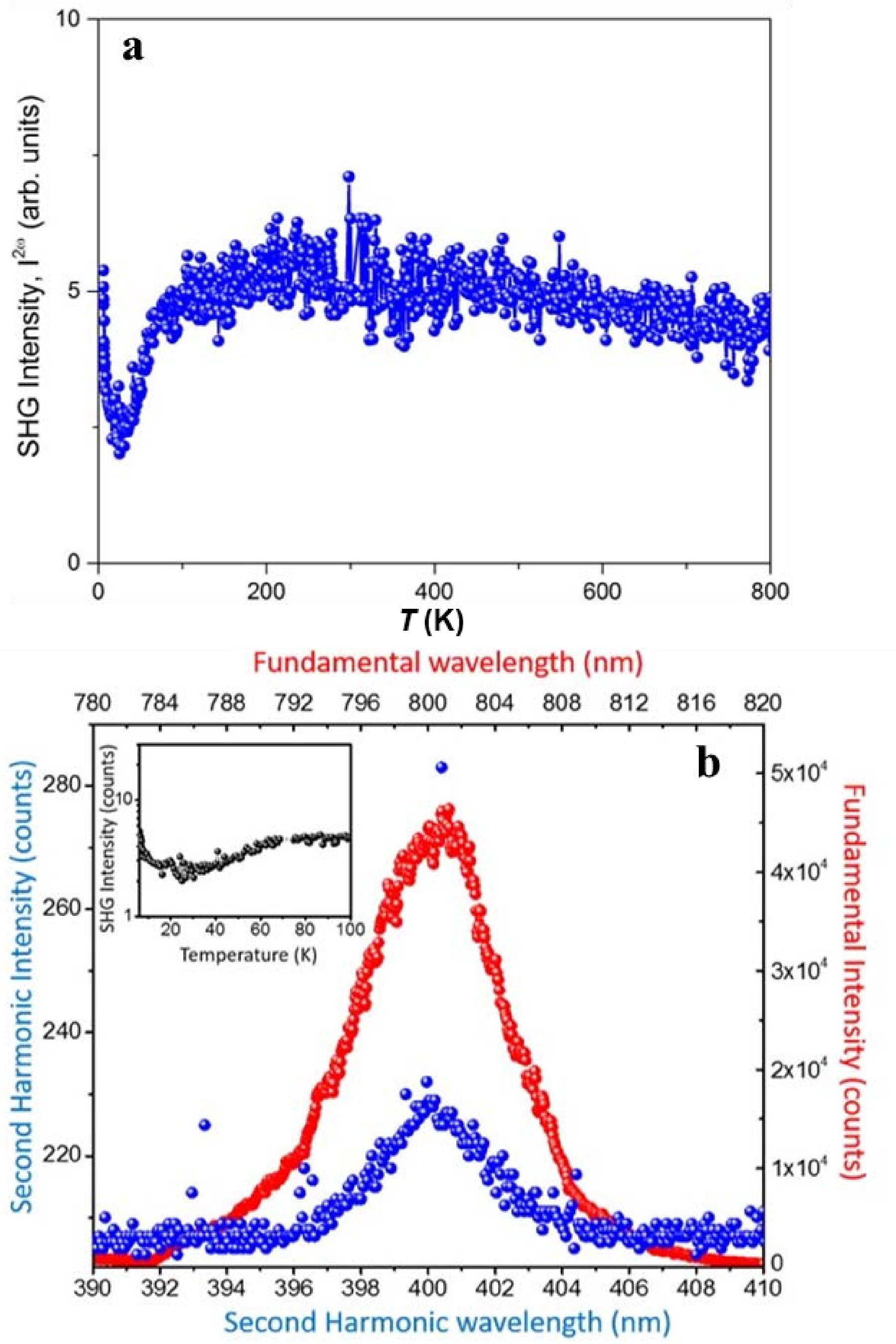

Supplementary Figure 15 │ SHG measurements on Mn_2MnWO_6. a SHG intensity (arb. units) *vs.* temperature (K). **b** SHG (400 nm) peak and fundamental wavelength (800 nm) peak in the spectroscopic data at 300 K. The inset shows an expanded view of the temperature scan in (**a**) that reflects the magnetostriction-polarization coupling.

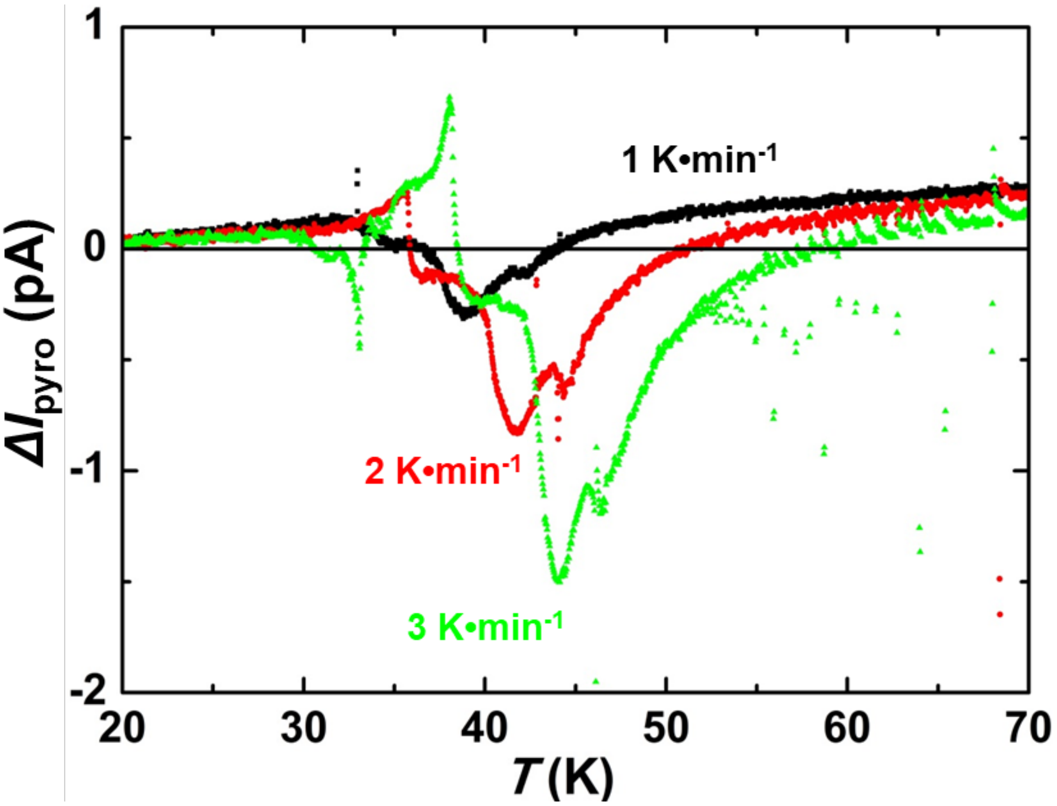

Supplementary Figure 16 | Temperature dependent pyrocurrent measurements in different warming rate of 1, 3, and 5 K•min^{-1}, spectively. These qualitatively confirm the expected scaling of the current with the rate of temperature change and thus exclude the possibility of ohmic, i.e. current related phenomena. (However the *T*-shift of the signature has to be attributed to the insufficient thermal coupling and thus denotes the experimental problems to determine e.g. a subtle shift of the feature in magnetic field as discussed above.) Also, the additional dielectric measurements now shown in **Fig. 4d** demonstrate that we indeed observe a change in polarization as this ac measurements are not subject to charge accumulation.

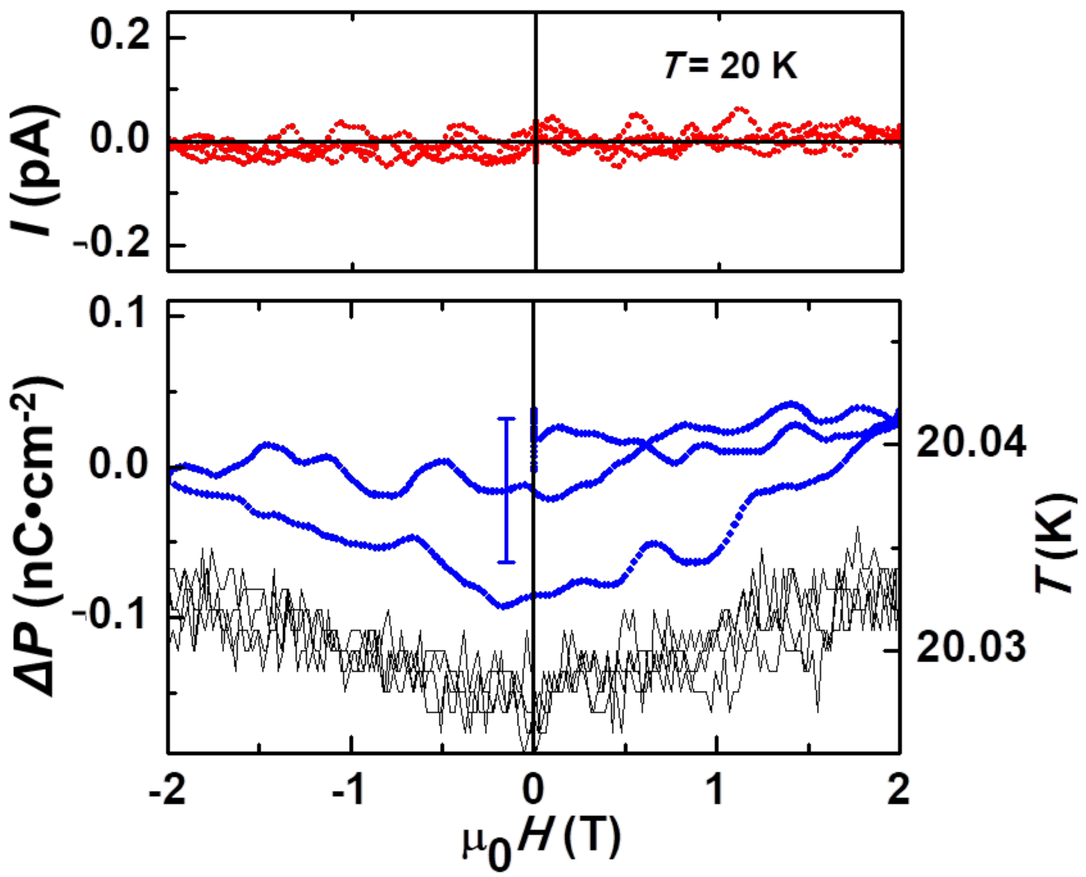

Supplementary Figure 17 | Magnetic-field-dependent polarization mesurements of Mn_2MnWO_6 at 20 K. The error bar is addressed on the bottom figure. The small *ΔP* response indicates weak magnetoelectric coupling in Mn_2MnWO_6

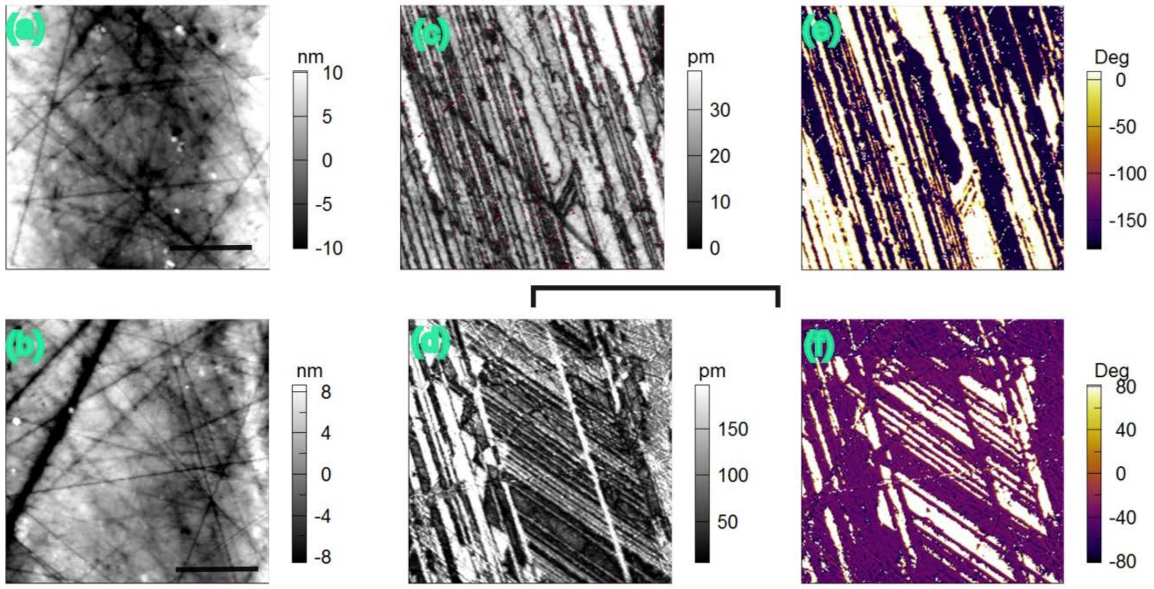

Supplementary Figure 18 | PFM images of Mn_2MnWO_6. (**a,b**) AFM topography maps from two different representative areas of the polished Mn_3WO_6 sample (scale bar = 2 μm) collected in contact mode AFM. Simultaneously captured piezoresponse (**c,d**) amplitude and (**e,f**) phase maps are also shown. Piezoresponse force microscopy (PFM) was performed using dual amplitude resonance tracking (DART). DART-PFM was implemented on a commercial SPM system (Asylum Research Cypher). Measurements were performed using Pt–Ir coated (Multi75E-G Budgetsensors) cantilevers. An excitation voltage of 6 Vp-p consisting of two drive frequencies above and below (~ 3 KHz) the contact resonance frequency (~ 340 KHz) were supplied to the tip during DART-PFM. The contact resonance (**c,d**) amplitude and (**e,f**) phase maps were determined from fitting the DART responses at both drive frequencies to a simple harmonic oscillator model. The PFM amplitude maps show typical ferroelectric domain structures which are independent from topography, and also clearly visible domain walls at which the amplitude drops close to 0. Further, PFM phase images show 180° phase offsets between domains, which are indicative of ferroelectric domains with opposite polarization.

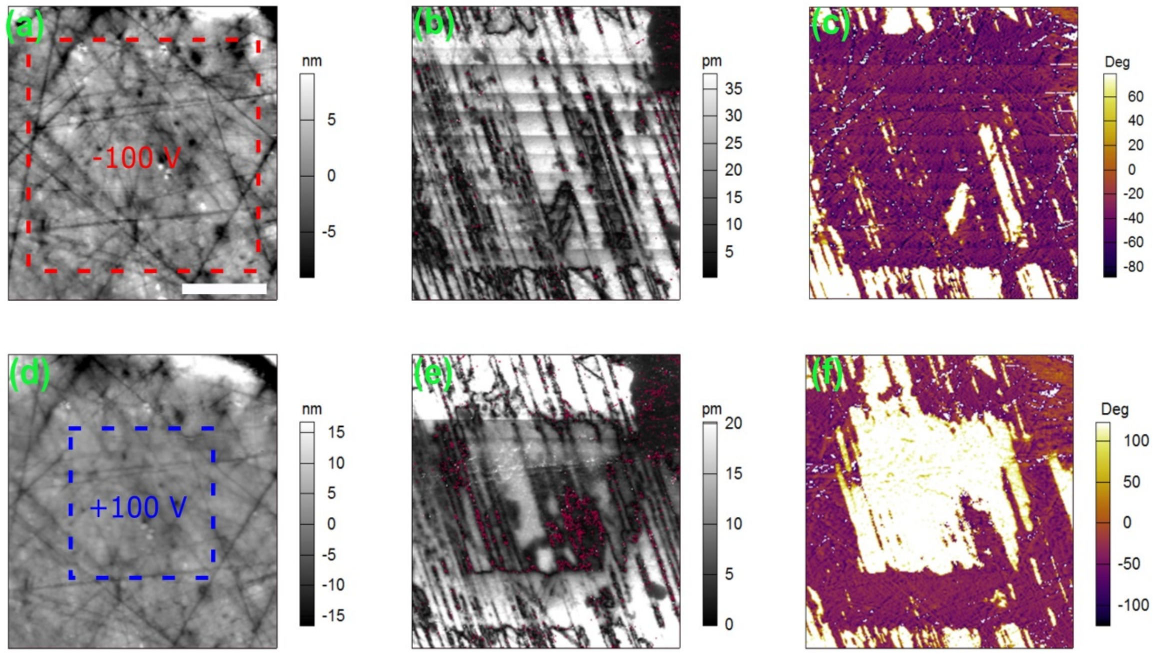

Supplementary Figure 19 | Topography and DART PFM images of Mn_2MnWO_6. (a,d) AFM topography (scale bar = 2 μm) and piezoresponse (**b,c**) amplitude and (**c,f**) phase maps after poling the pristine region shown in Supplementary Fig. 18a-c.PFM poling was performed using a commercial SPM system (Asylum Research Cypher) coupled with a high voltage amplitude (x10), and local poling was achieved by contact mode scanning using a constant force setpoint between tip and surface while controlling the voltage supplied the conductive tip. Piezoresponse amplitude and phase maps after poling a 4 x 4 μm region with -100 V and subsequently poling a smaller 2 x 2 μm with +100 V is shown in (**b,c**) and (**e,f**) respectively. Modification of the PFM domain structure between pristine and positively or negatively poled regions is clearly evident. The phase is shown to change by 180° between positively and negatively poled regions indicated controlled ferroelectric switching in this material by PFM.

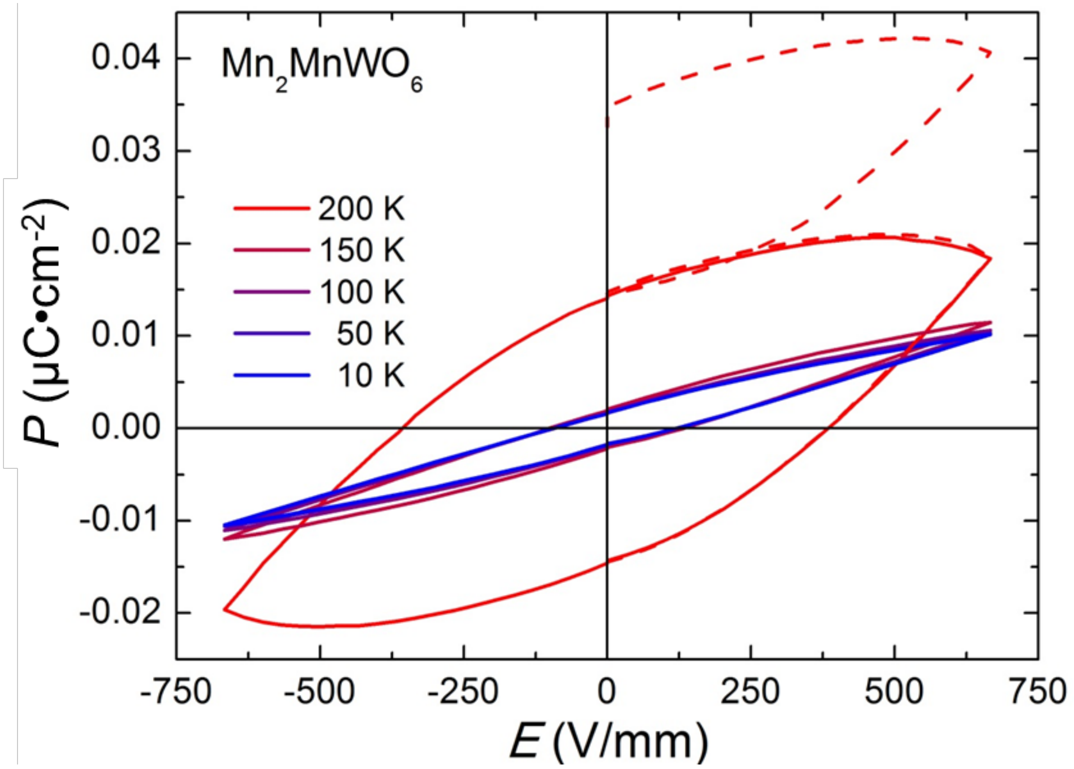

Supplementary Figure 20 | $P(E)$ loop measured on polycrystalline Mn_2MnWO_6 pellet between 10 and 150 K, showing very small switchable polarization (in the range of 0.005 μC•cm^{-2}). At 200 K the bulk conductivity dominates the polarization response of the sample: The dashed line is a double up-cycle of the electric field at the same temperature. The second up-cycle yields again a similar enhancement of polarization compared to the first which cannot be explained by the switching of intrinsic polarization but stems from ohmic contributions. However, at lower temperatures the residual conductivity of the sample decreases drastically and therefore this ohmic contribution vanishes leaving only the denoted remnant polarization.

Supplementary Tables

Compound	Synthesis	Structure	Magnetism	P_S (μC•cm^{-2})	Ref.
Mn_2FeNbO_6	1573 K, 7 GPa	LN, $R3c$	T_N = 90 K	32	[1]
Mn_2FeTaO_6	1573 K, 7 GPa	LN, $R3c$	T_N = 80 K	23	[1]
Mn_2FeMoO_6	1623 K, 8 GPa	NTO, $R3$	T_C = 337 K	68	[2]
Mn_2FeMoO_6	1623 K, 8 GPa[a]	OIL, $R3$	T_C = 229 K	55	[3]
Mn_2FeWO_6	1673 K, 8 GPa	NTO, $R3$	T_N = 75 K	59.5/67.8	[4]
Mn_2ScSbO_6	1523 K, 5.5 GPa	NTO, $R3$	T_C = 42 K	28.3	[5]
Mn_2MnWO_6	1673 K, 8 GPa	NTO, $R3$	T_N = 58 K	62.9/69	[6]
Ni_2NiTeO_6	1073 K, AP	NTO, $R3$	T_N = 52 K	0.33[b]	[7,8]
Ni_2ScSbO_6	1073 K, AP	NTO, $R3$	T_N = 60 K	7	[9]
Ni_2InSbO_6	1073 K, AP	NTO, $R3$	T_N = 76 K	7	[9]
Zn_2FeTaO_6	1623 K, 9 GPa	LN, $R3c$	T_N = 22 K	50	[10]
Sc_2FeMoO_6	Theory	NTO, $R3$	T_C = 923 K	7.1	[11]
Lu_2FeMoO_6	Theory	NTO, $R3$	T_C = 895 K	8.7	[11]

Supplementary Table 1 | Polar and magnetic oxides in $A_2BB'O_6$-type corundum family. The synthesis conditions, structure typs, magnetic properties, and polarization values are list for comparing.

Atom	Site	*x*	*y*	*z*	U_{iso} ×100 (Å^2)
Mn1	3*a*	0	0	0.2117(1)	1.80(7)
Mn2	3*a*	0	0	0.7172(1)	2.02(7)
Mn3	3*a*	0	0	0.4918(2)	1.69(7)
W	3*a*	0	0	0*	1.83(6)
O1	9*b*	0.2766(3)	-0.0134(3)	0.0933(1)	2.09(7)
O2	9*b*	0.6544(2)	-0.0485(3)	0.6009(1)	2.00(6)

Space group *R3* (146), *Z* = 3, *a* = 5.32323(3) Å, *c* = 14.0589(1) Å, volume = 345.01(1) Å^3; R_{wp} = 4.55%, R_p = 4.74%, χ^2 = 3.67. *W *z* factional coordinate fixed to define origin along [001].

Supplementary Table 2 | Structure parameters of Mn_2MnWO_6 at 290 K. The data are from combined refinements of SPXD NPD data.

$Mn1O_6$		$Mn3O_6$	
Mn1-O1 × 3<br>-O2 ×<br>3<br><Mn1-O><br>BVS<br>d_{Mn1}<br>Δ_{Mn1} (× 10^{-3})	2.247(2)<br>2.150(2)<br>2.198(2)<br>2.00<br>0.439<br>0.49	Mn3-O1 × 3<br>-O2<br>× 3<br><Mn3-O><br>BVS<br>d_{Mn3}<br>Δ_{Mn3} (× 10^{-3})	2.085(2)<br>2.308(2)<br>2.196(2)<br>2.09<br>0.308<br>2.58
$Mn2O_6$		WO_6	
Mn2-O1 × 3<br>-O2 ×<br>3<br><Mn2-O><br>BVS<br>d_{Mn2}<br>Δ_{Mn2} (× 10^{-3})	2.061(1)<br>2.377(2)<br>2.219(2)<br>2.06<br>0.517<br>5.07	W-O1 × 3<br>-O2 × 3<br><W-O><br>BVS<br>d_W<br>Δ_W (× 10^{-3})	1.999(1)<br>1.867(2)<br>1.933(2)<br>5.84<br>0.193<br>1.17
Mn1-W	2.976(2)	Mn2-Mn3	3.169(3)
O1-Mn1-O1<br>O2-Mn1-O2<br>O1-Mn1-O2	71.14(5)<br>107.42(5)<br>87.22(4)<br>88.00(5)<br>153.64(4)	O1-Mn3-O1<br>O2- Mn3-O2<br>O1- Mn3-O2	102.12(4)<br>80.67(4)<br>87.17(1)<br>87.85(6)<br>164.44(3)
O1-Mn2-O1<br>O2-Mn2-O2<br>O1-Mn2-O2	111.88(6)<br>77.88(4)<br>74.01(3)	O1-W-O1<br>O2-W-O2<br>O1-W-O2	81.65(6)<br>97.59(6)<br>87.83(4)

	90.03(2)<br><br>151.19(7)		91.89(6)<br><br>168.34(4)

Supplementary Table 3 | Crystallographic data in Mn_2MnWO_6 at 290 K from combined SPXD and NPD data refinements. Selected interatomic distances (Å), bond valence sums (BVS), atomic displacement distance (Å) from its octahedral center (d_M), octahedral distortion parameters (Δ), and bond angles (º) are shown.

**Atom**	**Site**	***x***	***y***	***z***	**U_{iso} × 100 (Å^2)**	***Mz*** **(μ_B)**	***Mxy*** **(μ_B)**
Mn1	6a	0	0	0.10664(8)	1.92(6)†	3.742(7)	1.87(1)
Mn2	6a	0	0	0.85807(9)	1.92(6)†	-1.871(4)	-3.94(2)
Mn3	6a	0	0	0.24404(9)	1.92(6)†	3.742(7)	1.87(1)
W	6a	0	0	0*	2.4(1)		
O1	18b	0.2886(3)	0.2765(4)	0.04743(6)	2.51(8)		
O2	18b	0.3690(4)	-0.0098(4)	0.13476(7)	1.34(6)		

* W *z* fractional coordinate fixed to define origin along [001]

† U_{iso} for Mn sites constrained to be equal to minimise correlation with Mn moments

Supplementary Table 4 | Crystallographic data for Mn_2MnWO_6 from Rietveld refinement using 5 K NPD data. Space group $R_I3(00g)t$ (hexagonal setting; assume *R*3*c* for nuclear structure), a = 5.31609(3) Å, c = 28.1388(4) Å, Volume = 688.69(1) Å^3; R_{wp} = 5.73%, R_p = 6.64%, χ^2 = 7.29.

Bond	Length (Å)	Bond	Length (Å)
Mn1 – O1	3 × 2.244(3)	Mn3 – O1	3 × 2.058(2)
Mn1 – O2	3 × 2.140(3)	Mn3 – O2	3 × 2.356(3)
Mn2 – O1	3 × 2.067(3)	W – O1	3 × 2.010(2)
Mn2 – O2	3 × 2.342(3)	W – O2	3 × 1.864(2)
Mn1 – W	3.001(2)	Mn2 – Mn3	3.209(2)

Supplementary Table 5 | Crystallographic data of Mn_3MnWO_6 at 5K. Selected bond lengths and distances from Rietveld refinement using 5 K NPD data are listed.

Supplementary Notes

Supplementary Note 1 | Polar and magnetic oxides in $A_2BB'O_6$-type corundum family.

Under specific experimental conditions, the formation of polar and magnetic corundum compounds can be governed by the electron configuration, spin structure, size and charge difference of cations, or a combination of these parameters. So far, only 14 polar and magnetic $A_2BB'O_6$-type corundum-related compounds have been reported (Supplementary Table 1). The AFM and pyroelectric LN-type $Mn^{2+}{}_2Fe^{3+}M^{5+}O_6$ (M = Nb, Ta) were prepared at 7 GPa[1]; first-principles calculations indicated that the second-order Jahn-Teller (SOJT) distortion effects of the d^0-configuration (Nb^{5+} and Ta^{5+}) favor the polar LN-type rather than the centrosymmetric ilmenite (IL, *R*-3) structure as observed in $Mn^{2+}{}_2Fe^{3+}Sb^{5+}O_6$ (ref. 12), considering the very similar ionic sizes of six-coordinated Nb^{5+} (0.64 Å), Ta^{5+} (0.64) and Sb^{5+} (0.60 Å)[13]. This finding has been corroborated by the synthesis of LN-type, AFM $Zn_2Fe^{3+}Ta^{5+}O_6$ at 9 GPa, which suggests a small switchable ferroelectric component at low temperature[10]. The polarized spin structure of the ferrimagnetic semiconductor $Mn^{2+}{}_2Fe^{3+}Mo^{5+}O_6$ (T_C ~ 337 K) allows significant energy lowering to stabilize in the NTO-type structure versus IL-type, despite the comparable effective ionic size (${}^{VI}r(Mo^{5+})$ = 0.61 Å) and charge difference of B and B' ions (2) compared to those in IL-type $Mn_2Fe^{3+}Sb^{5+}O_6$ and LN-type $Mn_2Fe^{3+}M^{5+}O_6$ (M = Nb and Ta)[1,12]. In the case of Ni_3TeO_6, the large charge difference between Ni^{2+} and Te^{6+} renders full ordering between both cations at B- and B'-sites. This cationic ordering is responsible for the polar, so-called NTO-type, structure (formally, $Ni^{2+}{}_2Ni^{2+}Te^{6+}O_6$)[7,8]. Similarly, in $Mn^{2+}{}_2Fe^{2+}W^{6+}O_6$, the high stability of octahedral W^{6+} (${}^{VI}r(W^{6+}$ = 0.60 Å) produces Fe^{2+} (${}^{VI}r(Fe^{2+}$ = 0.78 Å) oxidation state, and thus the large charge and size difference between W^{6+} and Fe^{2+} in B- and B'-sites favors the NTO-type structure[4,13,14]. The large effective ionic size differences between Sc^{3+}/Sb^{5+} (0.15 Å) and In^{3+}/Sb^{5+} (0.2 Å) seem to account for the cation-ordering and NTO-type structure in Ni_2ScSbO_6, Ni_2InSbO_6, and Mn_2ScSbO_6 (refs. 5,9) The ambient pressure form of

Mn_2FeMoO_6, which is obtained by a Mn/Fe cationic rearrangement when the HP NTO-structural polymorph is treated at unusually low-temperature (150 - 300 ^{o}C), is the only known OIL-type polar magnet in this family. The low-temperature transformation of HP-Mn_2FeMoO_6 is accompanied by dramatic physical property changes, including a ~ 120 K lower magnetic ordering temperature and three-orders of magnitude higher resistivity[3]. Above room-temperature multiferroic properties were predicted for ferrimagnetic $Zn_2Fe^{3+}Os^{5+}O_6$ (T_C ~ 394 K, P_S ~ 54.7 μC·cm^{-2}), which was predicted to adopt the LN-type structure, but was found to adopt the NTO-type structure (*R*3) with Fe^{3+} and Os^{5+} ordering over B- and B'-sites[15]. The NTO-type A_2FeMoO_6 (A = Sc, Lu) are predicted to be room-temperature ferroelectric (P_S = 7.1 and 8.7μC·cm^{-2} for A = Sc and Lu, respectively) and ferrimagnetic (T_C ~ 923 and 895 K, respectively) insulators, by first-principles calculations[11].

Supplementary Note 2 | X-ray absorption near edge spectroscopy (XANES).

Mn-K edge

The main edge features at 3-*d* transition metal K edges are dominated by 1*s* to 4*p* transition peak features, along with a step-continuum-onset-feature. The 4*p* features can be complicated by splitting into multiple features by the local atomic coordination/bonding and by admixed 3*d* configurations. Nevertheless these features manifest a chemical shift to higher energy with increasing valence, allowing the use of the K edge to chronicle the evolution of the transition metal valence state in compounds[1,2,4,10,14,16-24]. In Supplementary Fig. 3a the Mn-K main edge spectra for Mn_2MnWO_6 is compared to those of the isostructural Mn_2FeTO_6 (T = W and Mo) compounds which contain Mn only on the A-site. The spectra for a series of octahedrally coordinated Mn standard compounds are also shown. The $Mn^{2+}O$ standard on the other hand has edge sharing octahedra, which splits/broadens the main edge[24]. Inspection of the Mn_2MnWO_6 Mn-K spectrum suggests the presence of an A-site $Mn^{2+}{}_2FeWO_6$ like

component, however there is a clear excess of spectral intensity in the energy range labeled EI in Supplementary Fig. 3a.

The Mn-K main edge of Mn_2MnWO_6 should be a superposition of an A-site $Mn^{2+}{}_2FeTO_6$ like component (with a weight factor of 2/3) and a B-site Mn^{2+} component (with a weight factor of 1/3). To estimate the B-site component the Fe-K edge for the $Mn_2Fe^{2+}WO_6$[4] and $Mn_2Fe^{3+}MoO_6$[2] compounds (previously measured) were considered (see Supplementary Fig. 4). The shifting of the Fe-K edge spectra down in energy to the Mn-K edge was done using the difference in the peak energies of the NaCl structure $Mn^{2+}O$ and $Fe^{2+}O$ spectra (see Supplementary Fig. 3b). The estimated spectrum (est. in Supplementary Fig. 3b) is a superposition equal to the weighted sum of the components given by $\{2/3 \sim A^{2+} + 1/3 \sim B^{2+}\}$. For energies above 6.552 keV the estimated spectrum provides a remarkably good simulation of the Mn_2MnWO_6 Mn-K edge spectrum (particularly in view of the large Fe-K to Mn-K energy shifts). This energy range also includes the extra intensity EI region, noted above, and the spectral fall-off in intensity above it. This is the energy range in which the Mn^{2+} character of the B-site needed confirmation. The underestimated spectral weight of the est. spectrum at the steeply rising portion of the edge is not surprising in view of the substantial approximations implicit in the $2/3 \sim A^{2+}$ and $1/3 \sim B^{2+}$ component estimation process. Thus the Mn-K data are consistent with both the Mn^{2+} character at the A and B sites and with the very different spectral shapes at the two sites.

W-L_3 edge

The L_3 edges of transition metals (T) are dominated by very intense white line (WL) features due to dipole transitions into final d states as shown in Supplementary Figs. 5a and b[1,4,10,14,18-23]. The simplest signature of increasing T-valence states (decreasing d-count) is the chemical shift of the absorption edge to higher energy. Such an increasing-valence/increasing chemical shift of the centrum of the WL-feature has been observer in multiple

studies[1,4,14,18,20-23]. In Supplementary Fig. 5a the W-L_3 edge for Mn_2MnWO_6 is compared to those of a series of standard W compounds with varying *d*-configurations/valence states. The systematic chemical shift of the of the WL feature to higher energy, with increasing nominal W valence, is clear in the figure. Despite the bimodal A-B structure of WL-5*d* features in the A_2BWO_6 compounds their centrum chemical shift indicate a ~ d^0-W^{6+} configuration assignment (Supplementary Fig. 5c). Additional information on the W local environment can be gleaned from the W-L_3 edge XANES spectra on an energy range encompassing the prominent fine structure C-feature. This feature can be regarded as either the first of the ligand shell EXAFS oscillations (with multiple scattering) or as a continuum resonance due to the containment of the photoelectron by the filled shell ligand atoms. In either interpretation, the C-feature moves to higher energy with decreasing W-O bond lengths[4,18,22]. The close correspondence in energy of the C-feature of Mn_3WO_6 to that of the isostructural W^{6+} compound Mn_2FeWO_6 provides additional support for the identification of the W^{6+}, d^0 state in the former. The double perovskite compound W^{6+}, d^0 has a simpler corner sharing local environment, as opposed to the face and edge sharing in the Ni_3TeO_6 (NTO, *R*3) structure. Hence, the C-feature for this less distorted octahedron is more pronounced as well as indicating a shorter W-O bond length.

The bimodal A (t_{2g} related)/B (e_g related) structure of the L_3-WL features arises from the robust octahedral O-ligand coordination ligand field (LF), splitting of the *d*-states, into lower energy, 6X degenerate, t_{2g} and higher energy, 4X degenerate, e_g multiplets. This LF splitting is most clearly illustrated in the d^0 compound spectra shown in Supplementary Figs. 5a and b. The systematic filling of the t_{2g} orbitals with increasing 5*d*-orbital-count (decreasing hole-count) clearly leads to a systematic decrease of the A-feature (t_{2g}-hole coupled) intensity as is illustrated in Supplementary Fig. 5b for a series of compounds spanning d^0-d^4.[1,4,10,14,18-24] Here the T-L_3 spectra of the various elements have been displaced to nominally align the A-B

features. Thus the greater A-feature intensity, relative to the B-feature, for Mn_2MnWO_6 also strongly supports its ~ d^0-W^{6+} configuration assignment.

Supplementary Note 3 | Magnetic Properties. Referring to Supplementary Fig. 6, hysteretic 1st-order field induced transition below 30 K are defined by the inflection points in the *M*(*H*) plots. The metastability limits of these field induced transitions are indicated as dotted lines in the phase diagram in Supplementary Fig. 7. The lack of *M*(*H*) hysteresis at T = 50 K (see Supplementary Fig. 6) indicates a 2nd-order transition (as indicated in Supplementary Fig. 7), and lies very close to the thermal instability of the AFM low temperature phases. The region above the AFM phases has been labelled paramagnetic. It is worth noting that the T = 58 and 65 K curves show a disparate curvature, which suggest some field coupled magnetic correlations above the ordering temperature. The negative peak in the d*M*/d*T* plots in Supplementary Fig. 6 fall in this temperature range and are tentatively associated with local magnetic correlations precursive to the ordering temperature. The loci and peak widths of these negative d*M*/d*T* peaks are used to define a shaded region in the *H*-*T* phase diagram where magnetic correlations appear operative. These magnetic correlations couple to the external field and presumably involve field induced spin canting, however, the finite field neutron diffraction would be required to determine their true character. The fluctuation of the SHG response in Supplementary Fig. 15 at around T_N indicates the presence of spin-charge coupling, as shown in previous works.[25] This behavior is consistent with the magnetostriction-polarization coupling that is proposed.

Supplementary Note 4 | SHG effect. SHG experiments were performed on as-made pellets of Mn_2MnWO_6 in the reflection mode. The compound was observed to be SHG active between 6 K and 800 K (Supplementary Fig. 14a) as SHG intensity was significantly higher

than the background in this temperature range. As shown in Supplementary Fig. 14b, SHG intensity ($I^{2\omega}$) peak was observed at 400 nm wavelength in the spectroscopic data corresponding to the 800 nm fundamental wavelength that was irradiated on the pellet. The presence of the SHG peak confirms broken inversion symmetry in this compound. Following this experiment, temperature dependent SHG intensity was measured in a far-field SHG setup previously reported in Garten *et al.* [26].

Supplementary References

1 Li, M.-R. *et al.* Polar and Magnetic Mn_2FeMO_6 (M=Nb, Ta) with $LiNbO_3$-type Structure: High-Pressure Synthesis. *Angew. Chem. Int. Ed.* **52**, 8406-8410 (2013).

2 Li, M.-R. *et al.* Magnetic-Structure-Stabilized Polarization in an Above-Room-Temperature Ferrimagnet. *Angew. Chem. Int. Ed.* **53**, 10774-10778 (2014).

3 Li, M.-R. *et al.* Low-Temperature Cationic Rearrangement in a Bulk Metal Oxide. *Angew. Chem. Int. Ed.* **55**, 9862-9867 (2016).

4 Li, M.-R. *et al.* Mn_2FeWO_6: A New Ni_3TeO_6-Type Polar and Magnetic Oxide. *Adv. Mater.* **27**, 2177-2181 (2015).

5 Solana-Madruga, E. *et al.* High pressure synthesis of polar and non-polar cation-ordered polymorphs of Mn_2ScSbO_6. *Dalton Trans.* **44**, 20441-20448 (2015).

6 Ye, M. & Vanderbilt, D. Ferroelectricity in corundum derivatives. *Phys. Rev. B* **93**, 134303 (2016).

7 Becker, R. & Berger, H. Reinvestigation of Ni_3TeO_6. *Acta Crystallogr. Sect. E* **62**, i222-i223 (2006).

8 Oh, Y. S. *et al.* Non-hysteretic colossal magnetoelectricity in a collinear antiferromagnet. *Nat. Commun.* **5**, doi:10.1038/ncomms4201 (2014).

9 Ivanov, S. A. *et al.* Spin and Dipole Ordering in Ni_2InSbO_6 and Ni_2ScSbO_6 with Corundum-Related Structure. *Chem. Mater.* **25**, 935-945 (2013).

10 Li, M.-R. *et al.* Designing Polar and Magnetic Oxides: Zn_2FeTaO_6 - in Search of Multiferroics. *J. Am. Chem. Soc.* **136**, 8508-8511 (2014).

11 Song, G. & Zhang, W. Comparative studies on the room-temperature ferrielectric and ferrimagnetic Ni_3TeO_6-type A_2FeMoO_6 compounds (A = Sc, Lu). *Sci. Rep.* **6**, 20133, doi:10.1038/srep20133 http://www.nature.com/articles/srep20133#supplementary-information (2016).

12 Mathieu, R. *et al.* Magnetic order near 270 K in mineral and synthetic Mn_2FeSbO_6 ilmenite. *Appl. Phys. Lett.* **98**, 202505 (2011).

13 Shannon, R. Revised effective ionic radii and systematic studies of interatomic distances in halides and chalcogenides. *Acta Crystallogr. Sect. A* **32**, 751-767 (1976).

14 Retuerto, M. *et al.* Polar and Magnetic Layered A-Site and Rock Salt B-Site-Ordered Na*Ln*$FeWO_6$ (*Ln* = La, Nd) Perovskites. *Inorg. Chem.* **52**, 12482-12491 (2013).

15 Wang, P. S., Ren, W., Bellaiche, L. & Xiang, H. J. Predicting a Ferrimagnetic Phase of Zn_2FeOsO_6 with Strong Magnetoelectric Coupling. *Phys. Rev. Lett.* **114**, 147204 (2015).

16 Croft, M. *et al.* Systematic Mn d-configuration change in the $La_{1-x}Ca_xMnO_3$ system: A Mn K-edge XAS study. *Phys. Rev. B* **55**, 8726-8732 (1997).

17 Mandal, T. K. *et al.* La_2MnVO_6 double perovskite: a structural, magnetic and X-ray absorption investigation. *J. Mater. Chem.* **19**, 4382-4390 (2009).
18 Popov, G., Greenblatt, M. & Croft, M. Large effects of A-site average cation size on the properties of the double perovskites $Ba_{2-x}Sr_xMnReO_6$: A d^5-d^1 system. *Phys. Rev. B* **67**, 024406 (2003).
19 Mandal, T. K., Poltavets, V. V., Croft, M. & Greenblatt, M. Synthesis, structure and magnetic properties of $A_2MnB'O_6$ (A=Ca, Sr; B'=Sb, Ta) double perovskites. *J. Solid State Chem.* **181**, 2325-2331 (2008).
20 Li, M.-R. *et al.* Giant Magnetoresistance in the Half-Metallic Double-Perovskite Ferrimagnet Mn_2FeReO_6. *Angew. Chem. Int. Ed.* **54**, 12069-12073 (2015).
21 Kayser, P. *et al.* Crystal and Magnetic Structure of Sr_2BIrO_6 (B = Sc, Ti, Fe, Co, In) in the Framework of Multivalent Iridium Double Perovskites. *Eur. J. Inorg. Chem.* **2015**, 5027-5038 (2015).
22 Lin, Q., Greenblatt, M. & Croft, M. Evolution of structure and magnetic properties in electron-doped double perovskites, $Sr_{2-x}La_xMnWO_6$ ($0 \leqslant x \leqslant 1$). *J. Solid State Chem.* **178**, 1356-1366 (2005).
23 Retuerto, M. *et al.* High magnetic ordering temperature in the perovskites $Sr_{4-x}La_xFe_3ReO_{12}$ (x = 0.0, 1.0, 2.0). *J. Solid State Chem.* **194**, 48-58 (2012).
24 Li, M.-R. *et al.* Synthesis, crystal structure, and properties of $KSbO_3$-type $Bi_3Mn_{1.9}Te_{1.1}O_{11}$. *J. Solid State Chem.* **197**, 543-549 (2013).
25 Ramirez, M. O. *et al.* Magnon sidebands and spin-charge coupling in bismuth ferrite probed by nonlinear optical spectroscopy. *Phys. Rev. B* **79**, 224106 (2009).
26 Garten, L. M. *et al.* Relaxor Ferroelectric Behavior in Barium Strontium Titanate. *J. Am. Ceram. Soc.* **99**, 1645-1650 (2016).
